# Observing abilities of satellite-tagged sea turtles: comparison of reconstructed temperature profiles with ocean model data in the Adriatic and Ionian Seas

**DOI:** 10.1038/s41598-026-46945-5

**Published:** 2026-04-01

**Authors:** Daniele Piazzolla, Simone Bonamano, Carla Cherubini, Viviana Piermattei, Marco Marcelli, Giacomo Marzano, Francesco De Franco, Emanuela Clementi, Ivan Federico, Giovanni Coppini, Rosalia Maglietta

**Affiliations:** 1 CMCC Foundation - Euro-Mediterranean Center on Climate Change, Lecce, Italy; 2 Laboratory of Experimental Oceanology and Marine Ecology, Department of Ecological and Biological Sciences (DEB), Università degli Studi della Tuscia, Civitavecchia, Italy; 3 Department of Electrical and Information Engineering, Polytechnic of Bari, Bari, Italy; 4 Institute of Intelligent Industrial Technologies and Systems for Advanced Manufacturing, CNR-STIIMA, Bari, Italy; 5 Consorzio di gestione di Torre Guaceto, Carovigno, Brindisi, Italy

**Keywords:** Animal-borne sensors, satellite tag, sea turtles, *C. caretta*, statistical models, numerical models, Climate sciences, Ecology, Ecology, Ocean sciences

## Abstract

In situ and satellite-based oceanographic data are essential to understanding marine dynamics. In this study, we explore the ability of seawater temperature profiles along the water column, reconstructed from data collected by satellite-tagged loggerhead sea turtles, to capture ocean thermal structures. Temperature and depth data collected by seven loggerhead turtles (*Caretta caretta*) equipped with satellite tags in the Adriatic and northern Ionian Seas were compared with Copernicus Marine model products. Discrepancies between observed and CMEMS MedFS data primarily occur at intermediate (15 to 50 m) and greater depths (50 to 100 m), especially during summer and winter seasons, when stratification and limited deep-water observations reduce accuracy. These differences were most pronounced in dynamically complex areas such as the Western Adriatic Coastal Current (WACC) region and in the northern and middle Adriatic Seas, where fine-scale coastal processes and intense winter cooling challenge the resolution of both the CMEMS MedFS data and the animal-borne sensors. Although limited in sample size, the dataset offers a valuable opportunity to evaluate the additional observational insights provided by animal-borne sensors in challenging oceanographic environments, emphasizing the complementary role of turtle-borne observations within existing monitoring networks.

## Introduction

Environmental surveys using animal-borne sensors can provide both important insights into oceanographic conditions in hard-to-sample regions and vast data collection capabilities (e.g., unlimited casts per day)^1–5^. In addition to their well-known usefulness for the study of animal behavior (e.g., large-scale oceanic movements, fine-scale coastal movements, dive characteristics, habitat selection^6–9^, interest in the potential of data collected by animal-borne sensors for ocean observing systems and modeling is growing globally^4,10–14^.

Animal-borne sensors have been applied in various contexts worldwide. For instance, they have been used to better estimate surface mixed layer properties and circulation patterns within and south of the Antarctic Circumpolar Current^15^, to improve temperature and salinity measurements in the Southern Ocean^16^, and to refine representations of mesoscale eddies and frontal variations in the Kuroshio-Oyashio Confluence region^17^. More recent applications include validating models of sea ice drift patterns^18^ and enhancing the West Scotland Coastal Ocean Modelling System (WeStCOMS)^19^.

It is essential to acknowledge that datasets derived from animal-borne sensors exhibit interspecific variability, potentially resulting in differing uncertainties in the measurement of oceanographic parameters. Such variability is predominantly influenced by species-specific diving behaviors and habitat utilization patterns, which modulate the spatial and temporal representativeness of the collected oceanographic observations^12^.

The limited availability of direct observational data^20^, which are essential for studying physical and ecosystem variability and for improving numerical models used both at local and large-scales remains a major obstacle in monitoring and predicting ocean states and processes. This issue is mainly related to the remoteness of many locations^20^, the generally high costs of traditional oceanographic measuring platforms^21^, and the limited accessibility of user-friendly, low-cost marine monitoring instrumentation^22^. To address this issue, developing and using cost-effective technologies and effective observing systems is fundamental. These observing systems should also incorporate specific measurement platforms capable of providing adequate spatiotemporal resolution and coverage to meet the needs of monitoring plans^23^.

In the Mediterranean Sea, measurements from animal-borne sensors have predominantly focused on collecting geolocation and temperature data^24–26^. Among marine animal species, sea turtles are well-suited to satellite tagging and sensor deployment worldwide especially because of the good attachment surface provided by the carapace^6^. In the Mediterranean Sea, several initiatives have involved tagging sea turtle species to collect both ecological and environmental data^7,24–26^.

This study aims to assess the ability of temperature profiles along the water column, reconstructed from data collected by tagged loggerhead sea turtles (*Caretta caretta*, Linnaeus, 1758), to capture ocean thermal structures.

To this end, the reconstructed profiles (hereafter referred to as “observed data”) were compared with seawater temperature data from the Copernicus Marine Service Mediterranean Analysis and Forecasting System (CMEMS MedFS data, MEDSEA_ANALYSISFORECAST_PHY_006_013, product DOI: 10.48670/mds-00359) in the Adriatic and northern Ionian waters. In addition, the observed data were compared with ARGO float observations when sufficient spatial and temporal overlap allowed the same dynamical processes to be sampled.

For the last decade, the CMEMS has been providing users with high-quality regional ocean analysis that are regularly updated and extended, reinforcing their role as a critical tool in both scientific research and applied ocean monitoring. CMEMS provides an ocean monitoring and forecasting service delivering satellite and in-situ observations as well as numerical model data to describe the global oceans^27^ and six European regional seas. CMCC develops and operationally delivers the Mediterranean Physical Forecasting System (MedFS) within the Mediterranean Monitoring Forecasting Center (MED-MFC). The CMEMS model and in-situ and satellite datasets are extremely useful for studying anthropogenic and climate change-induced effects on seas and oceans^28–30^ and to monitor the state of health of key species^31–35^.

## Results

**Fig. 1:**
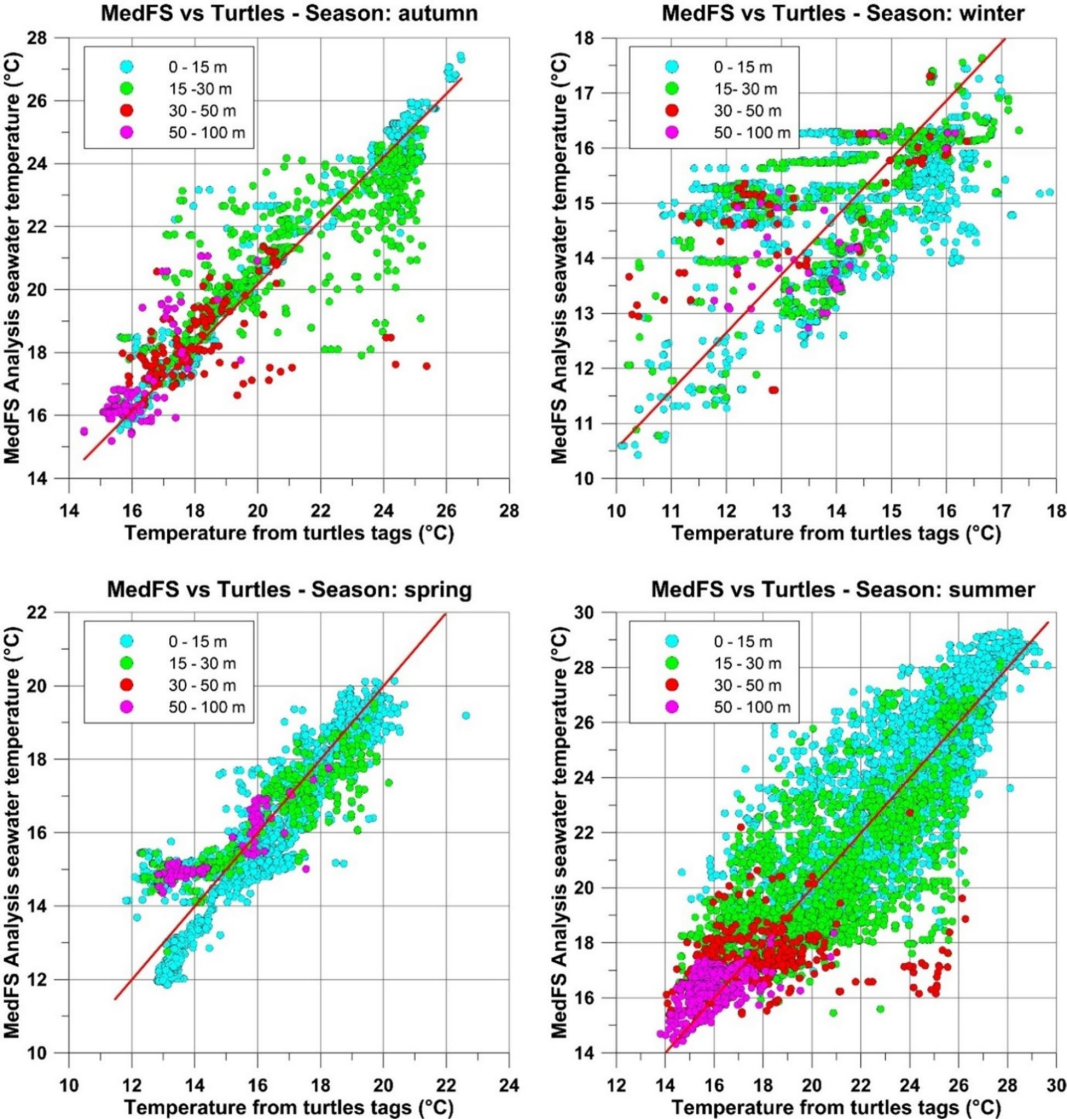
Scatterplots illustrating the relationship between observed and CMEMS MedFS seawater temperature data across the four seasons. The x-axis represents the observed temperature, recorded by sensors attached to sea turtles, while the y-axis shows the modeled temperature data. Circles are color-coded by depth, and a red line indicating the linear regression best fit is drawn in each plot.

The scatterplots in Fig. 1 show agreement between observed and CMEMS MedFS data from July 2020 to February 2022. The data points are largely aligned along the diagonal, suggesting a linear relationship between the two datasets, most evident in spring.

Table 1 summarizes the Pearson correlation coefficients (r) between observed and CMEMS MedFS data across different seasons and depth ranges, reporting only statistically significant results (*r* > 0.6, p-value < 0.05). Spring exhibited the highest correlations at all depths (*r* ≥ 0.87), indicating a match between observed and CMEMS MedFS data. Specifically:

**Table 1:** The table summarizes the correlation coefficients (r) between sea temperature and seasonal variations across different depth ranges.

Season	Depth (m)	Correlation Coefficient (*r*)	*N*. obs.
Winter	15-30	0.66	590
	30-50	0.61	88
	50-100	0.61	67
Spring	0-15	0.9	4630
	15-30	0.9	460
	30-50	0.87	142
	50-100	0.88	119
Summer	0-15	0.86	10,621
	15-30	0.68	1865
Autumn	0-15	0.98	2439
	15-30	0.85	881
	50-100	0.76	180

For each season (winter, spring, summer, and autumn) and depth interval (0–15 m, 15–30 m, 30–50 m, and 50–100 m), the correlation coefficients and the number of observations (N. obs.) are reported. Only depth ranges with *r* > 0.6 are included, and all reported r values are statistically significant (*p* < 0.05).

- Surface waters (0–15 m) showed positive, significant correlations in spring, summer, and autumn (*r* ≥ 0.86).
- In the 15–30 m depth range, statistically significant correlations were found in all seasons (*r* ≥ 0.66).
- At 30–50 m, the data analyzed showed significant correlations only during winter and spring, with *r* = 0.61 and *r* = 0.87, respectively. This may be attributed to the pronounced vertical temperature gradient that forms in summer and autumn, as surface heating intensifies and stratification strengthens in the upper ocean layers.
- At 50–100 m depth range, statistically significant correlations were found in winter (*r* = 0.61), spring (*r* = 0.88), and autumn (*r* = 0.76).

**Fig. 2:**
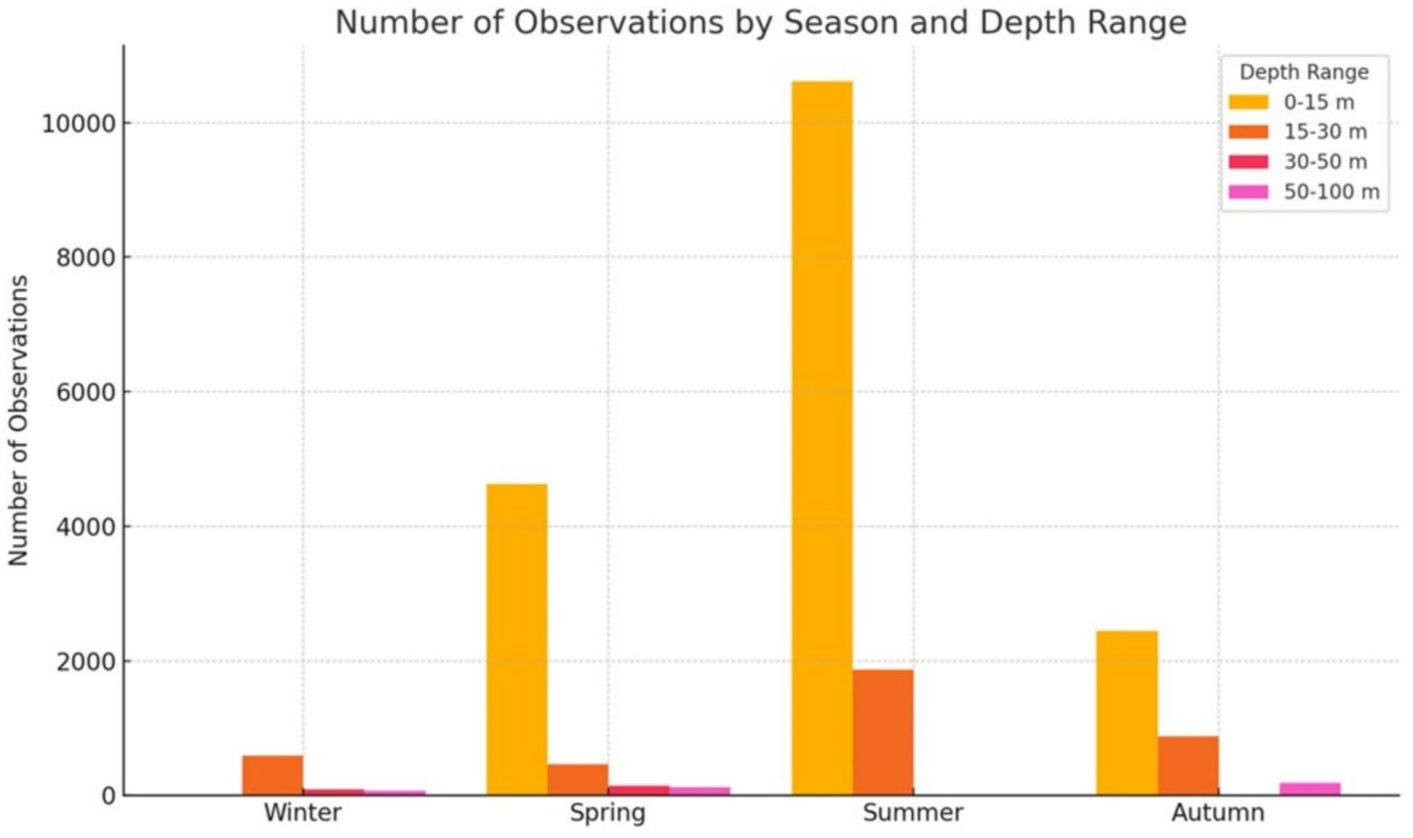
Number of observations across seasons. The colors reflect the four considered depth ranges (0–15 m, 15–30 m, 30–50 m, 50–100 m).

The number of observations (N. obs. in Tables 1 and 2) varied considerably, with more data available in summer and spring than in winter, and the largest number of observations collected in the upper water column (Fig. 2). This uneven distribution may have influenced the robustness of the estimates.

**Table 2:** The dataset used in this study covers the period from July 2020 to February 2022.

Tag ID	First Data	Last Data	*N*. obs.
202,778	23-07-2020	10-03-2021	141
202,779	29-07-2020	22-10-2021	3693
202,780	14-11-2020	15-02-2022	833
202,781	24-12-2020	27-08-2021	1039
202,782	04-01-2021	07-09-2021	1075
202,783	13-03-2021	16-10-2021	277
202,784	12-03-2021	16-09-2021	2121

The table reports tag IDs, the first transmitted in-water data (“first data”), the last transmitted data (“last data”), and the number of observations recorded for each tag.

**Fig. 3:**
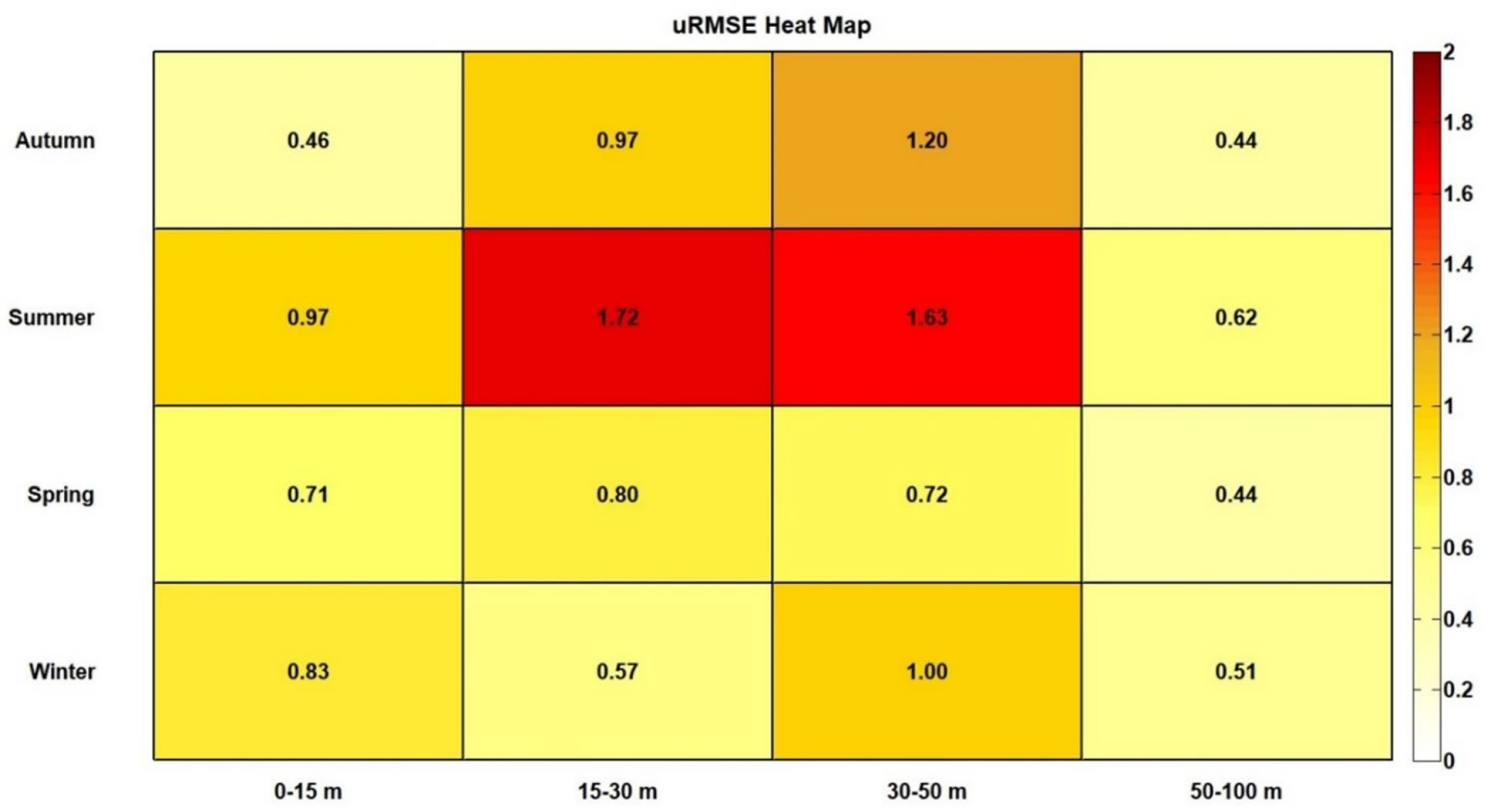
Heatmap of unbiased Root Mean Square Error (uRMSE) for temperature (°C) across different depths (x-axis) and seasons (y-axis). The values represent seasonal averages (y-axis) for specific vertical intervals (x-axis). The color scale represents uRMSE values, with dark red indicating higher errors and light yellow to white indicating lower errors.

**Fig. 4:**
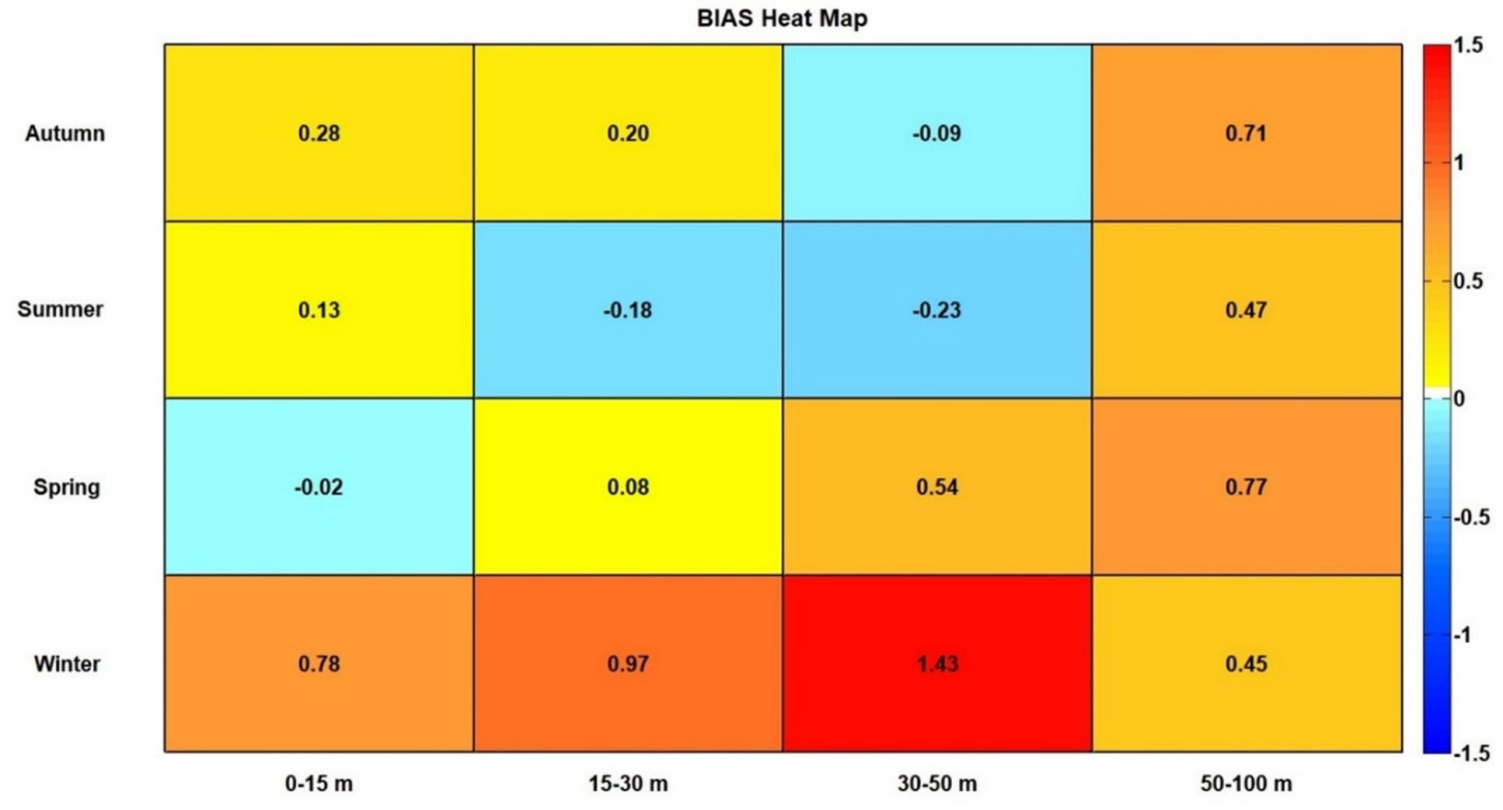
Heatmap of temperature BIAS (°C) (model minus observations) across depths (x-axis) and seasons (y-axis). The values represent seasonal averages (y-axis) for specific vertical intervals (x-axis). The color scale represents BIAS values, with reddish shades indicating positive values and bluish shades indicating negative values.

**Fig. 5:**
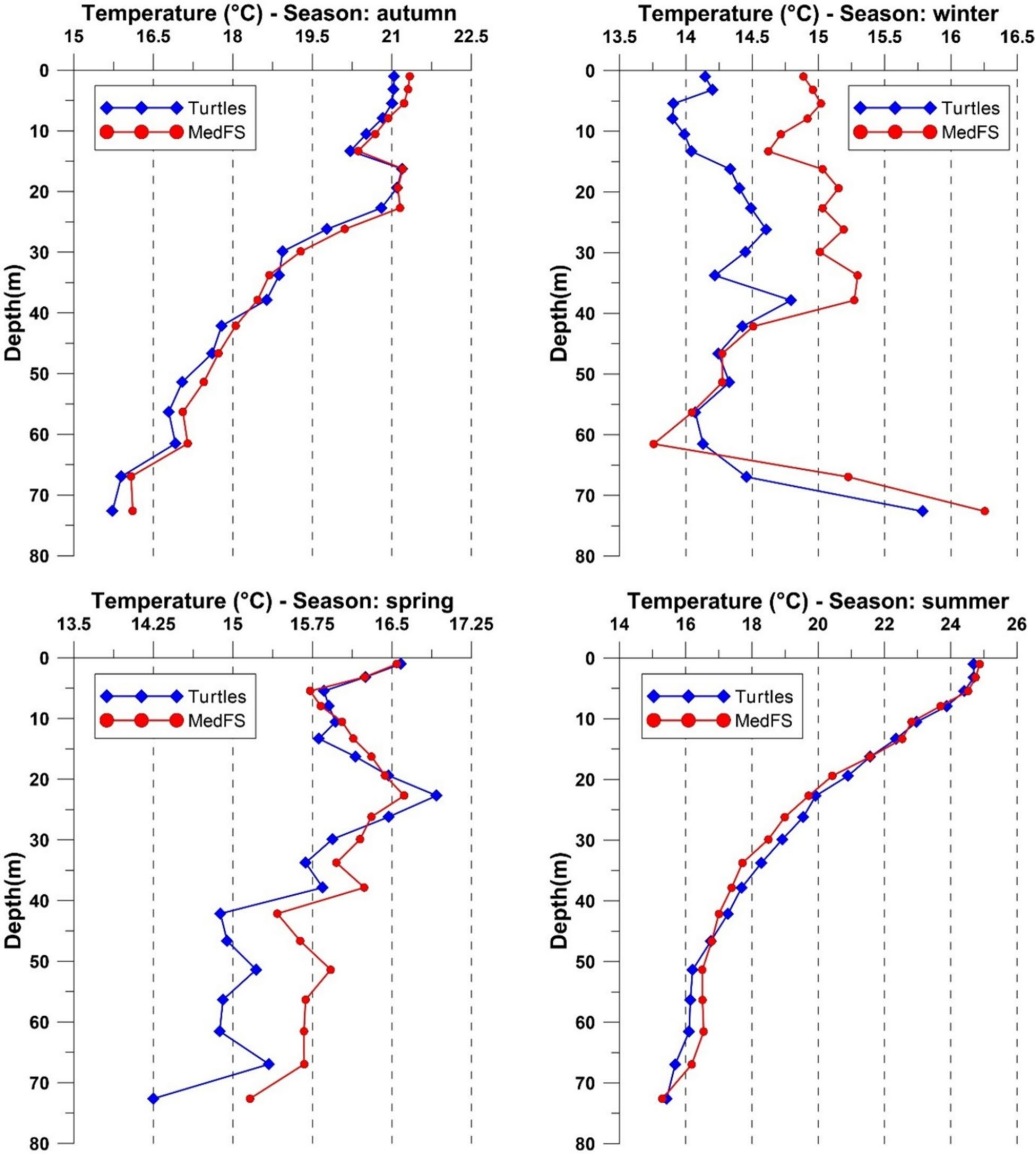
Comparison of observed (blue) and CMEMS MedFS (red) mean temperature-depth profiles across the four seasons. Vertical profiles were obtained by temporally averaging (at the seasonal scale) the temperature within the vertical layers considered in the CMEMS MedFS model.

**Fig. 6:**
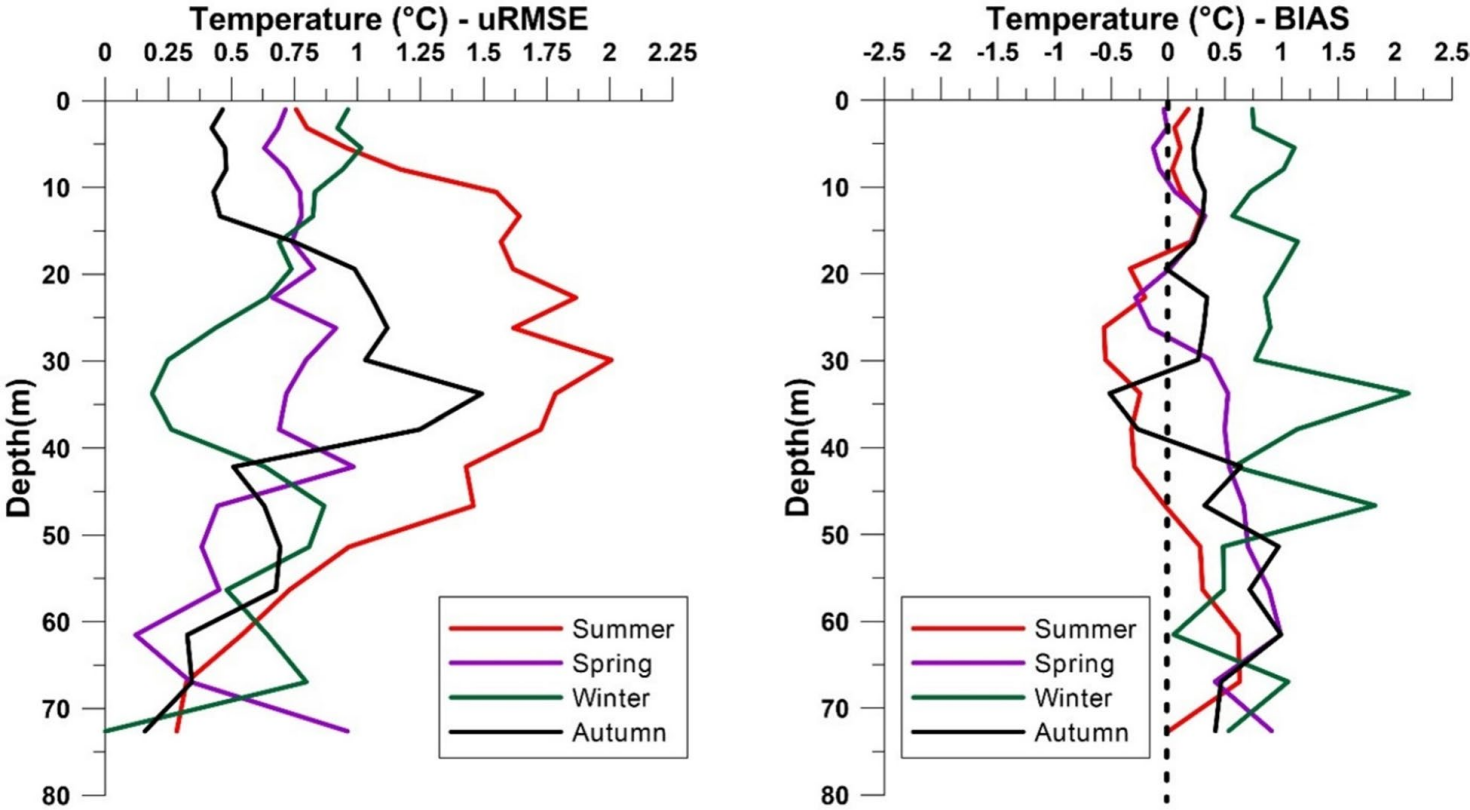
Vertical profiles of temperature BIAS (left) and uRMSE (right, °C) between observed and CMEMS MedFS data across seasons. Vertical profiles were obtained by temporally averaging (at the seasonal scale) BIAS and uRMSE values within the vertical layers considered in the CMEMS MedFS model.

The uRMSE and BIAS highlighted differences between observed and CMEMS MedFS data. In the uRMSE heatmap (Fig. 3), the 15–30 m and 30–50 m depth intervals showed the most significant differences during the summer and autumn. Conversely, observed data agreed with CMEMS MedFS data in the 0–15 m and 50–100 m depth ranges across all seasons. The BIAS heatmap (Fig. 4) revealed discrepancies exceeding 0.7 °C during the winter down to 50 m depth, as well as in the deepest layer (50–100 m) during autumn and spring. BIAS values near zero were observed down to 50 m, particularly in the autumn and summer seasons. In more detail, the comparison of seasonal temperature profiles between the two datasets along the water column (Fig. 5) showed consistent vertical patterns, demonstrating that the observed data successfully captured the physical processes occurring throughout the water column. The uRMSE vertical profiles (Fig. 6, left) indicated that the greatest differences between the datasets occurred during the summer, while the vertical trend of the BIAS (Fig. 6, right) showed that observed data exhibited the largest underestimations relative to CMEMS MedFS data during the winter season.

The extensive spatial and temporal coverage of the tags allowed us to assess the effectiveness of observed data in capturing vertical temperature variations across four sub-regions of the Adriatic Sea^42,43^: the Western Adriatic Coastal Current (WACC) region, located between the Po Delta and the Strait of Otranto along the Italian coast; the northern Adriatic (NA) region, defined by the 100 m bathymetry north of the Jakuba Pit; the Middle Adriatic (MA) region, with the southern boundary across the basin starting from the Gargano Peninsula; the Southern Adriatic (SA) region. Observations from the northern Ionian Sea were excluded from this analysis due to their uneven distribution throughout the year, being concentrated mainly in the autumn and spring seasons.

**Fig. 7:**
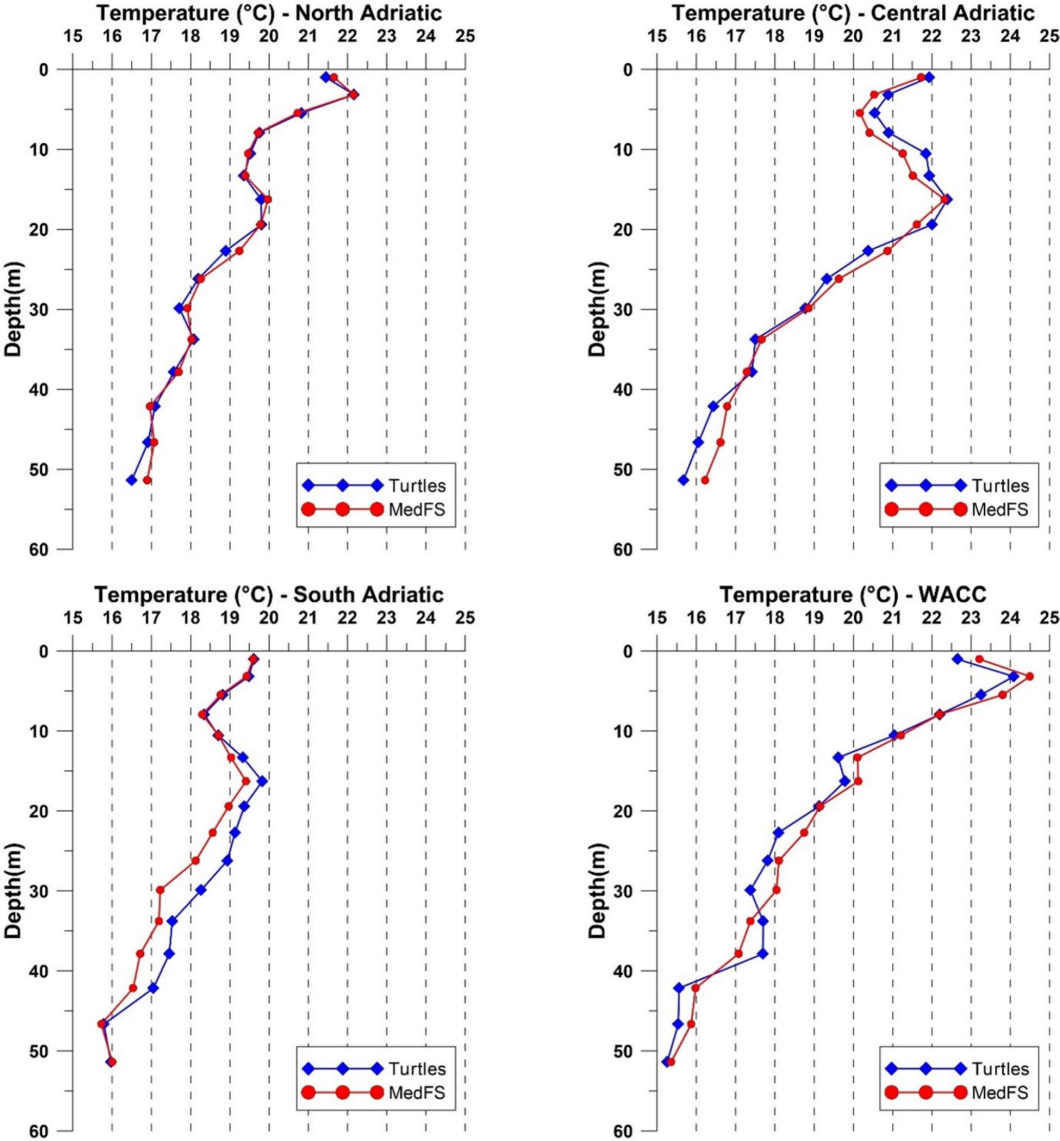
Comparison of mean temperature depth profiles between observed (blue) and CMEMS MedFS (red) data in the four sub-regions of the Adriatic Sea considered in this study. Profiles were obtained by spatially averaging all data within each subregion and temporally averaging the temperature within the vertical layers considered in the CMEMS MedFS model.

**Fig. 8:**
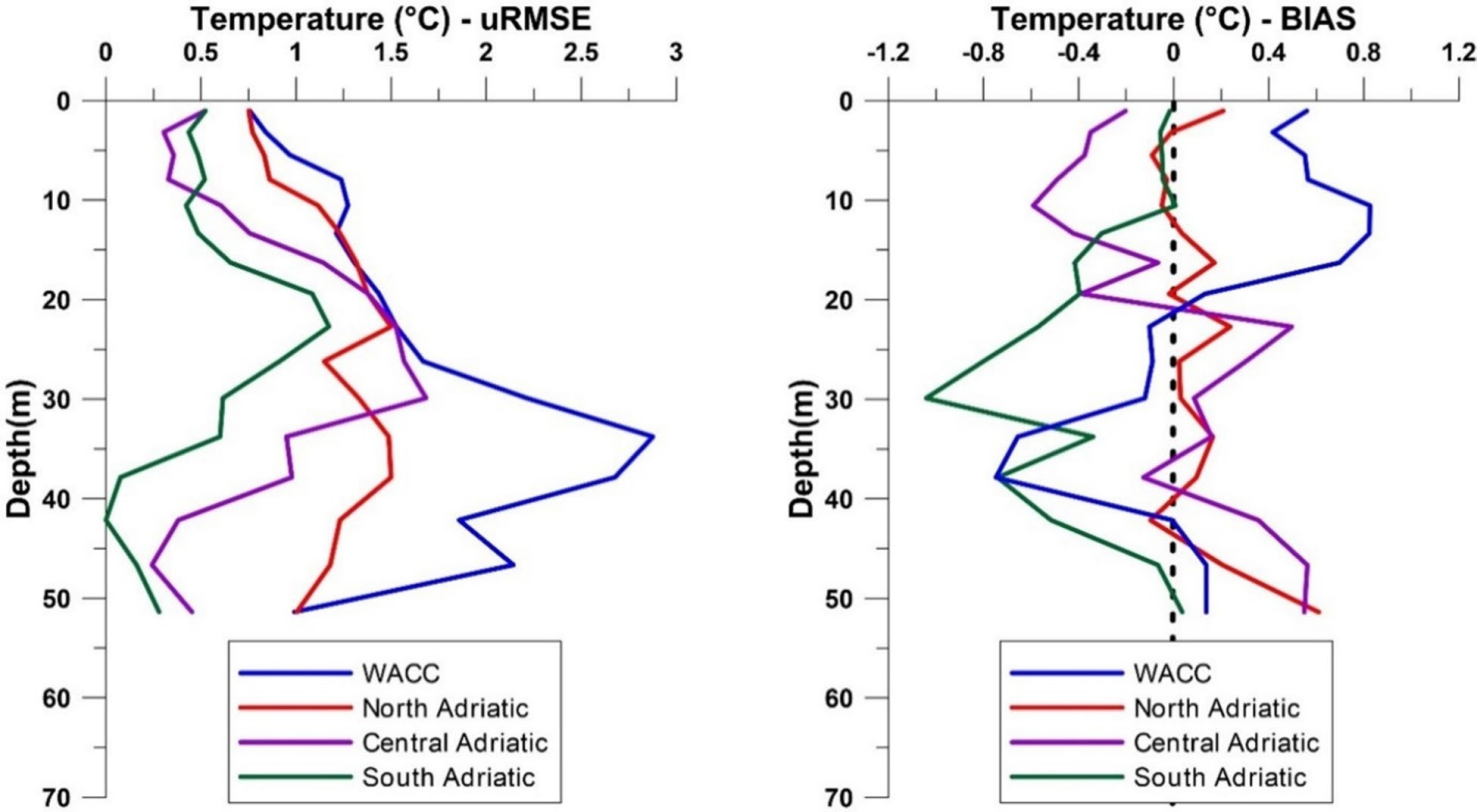
Vertical profiles of temperature BIAS (left) and uRMSE (right, °C) between observed data and CMEMS MedFS products for the different Adriatic sub-regions. Profiles were obtained by spatially averaging all data within each subregion and temporally averaging BIAS and uRMSE values within the vertical layers considered in the CMEMS MedFS model.

In the SA region, observed data were generally higher than MedFS values, with RMSE never exceeding 1.2 °C (Fig. 7). However, intermediate layers showed notably high BIAS, with observed data consistently higher than CMEMS MedFS ones (Fig. 8). In the MA and NA regions (Fig. 7), the surface layer was well reproduced (uRMSE < 1 °C), but intermediate layers showed higher variability, with uRMSE peaking near 1.5 °C (Fig. 8). BIAS was small in NA (± 0.2 °C) but larger in MA (± 0.4 °C) (Fig. 8). Along the WACC, uRMSE values were highest, especially at intermediate and deep layers (approaching 3 °C, Fig. 8). BIAS was also elevated, with positive values near the surface and negative at depth, indicating a strong mismatch between observed and CMEMS MedFS data (Fig. 8).

**Fig. 9:**
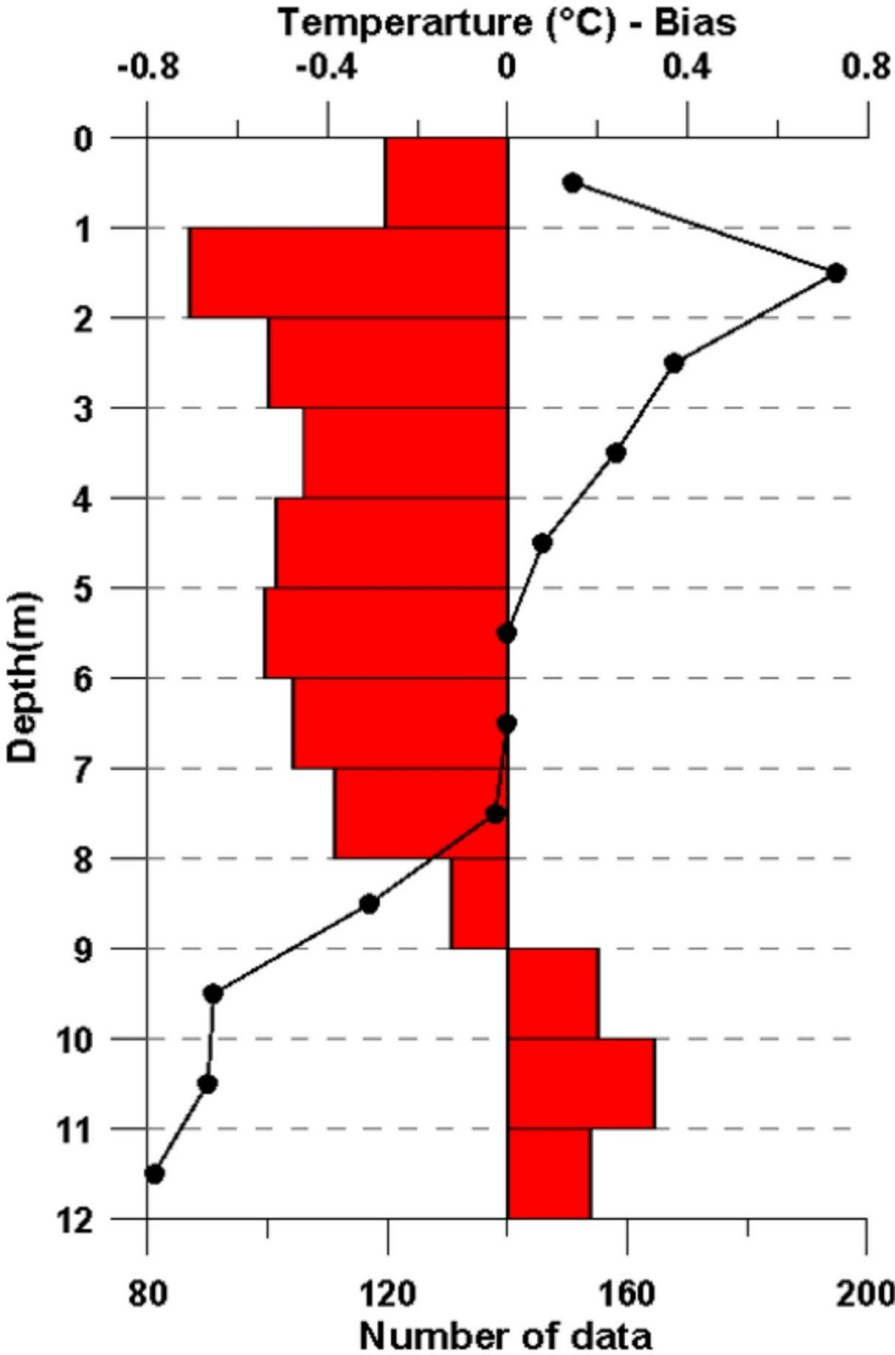
Temperature (^o^C) BIAS (red bars) calculated at 1 m intervals between ARGO data and satellite-tagged turtle data in the Adriatic Sea. The number of observations used for the comparison in each depth layer is also shown (black line).

To further assess the ability of observed data to capture ocean thermal structures, we conducted an upper-ocean consistency check using ARGO float measurements collected within the same geographic region and time frame (Fig. 9). BIAS values remained within ± 1 °C, with the greatest discrepancies between the two datasets occurring from the surface down to 8 m depth.

## Discussion

Animal-borne sensors have been increasingly used in recent years because they provide valuable oceanographic data from hard-to-reach areas and offer a cost-effective way to acquire continuous and essential information on oceanic variables^17^.

In this study, the ability of temperature profiles along the water column, reconstructed from data collected by satellite-tagged loggerhead sea turtles, to capture ocean thermal structures was assessed in the Adriatic and northern Ionian Seas. This assessment primarily relied on CMEMS MedFS data, which provide adequate spatial (~ 4 km) and temporal (1 day) coverage. Furthermore, it was possible to compare the observed data with ARGO float data for the upper layers of the water column over a short time interval in the southern Adriatic Sea.

*C. caretta* specimens were chosen as hosts for the sensors due to their ability to travel long distances in hard-to-access environments, thus offering long-term data on environmental variables such as water temperature, salinity, and depth, with minimal impact on their natural behavior; this makes them ideal for ecological and oceanographic research^36,37^.

The statistical indices used in this study (Pearson correlation coefficient, RMSE, and BIAS) showed that observed data were able to capture the vertical temperature patterns in the study area. The correlation analysis revealed that the agreement between observed, and CMEMS MedFS data was highly dependent on depth, season, and the dynamic of the areas. Surface waters (0–15 m depth range) showed the strongest correlation, especially in spring (*r* = 0.90) and autumn (*r* = 0.98), indicating that observed data effectively capture thermal dynamics in these periods. However, at intermediate depths (15–30 m), the correlation weakened in summer (*r* = 0.68), reflecting the difficulty in accurately assessing the thermocline displacement during this season. At larger depths (30–50 m and 50–100 m), the correlations were generally lower, underscoring the challenges in capturing deeper water dynamics.

The uRMSE analysis further confirmed the consistency of vertical temperature patterns between the two datasets, demonstrating the ability of observed data to capture key physical processes such as air-sea heat exchange and vertical mixing. However, during winter, despite an accurate reproduction of the vertical temperature profile, large positive BIAS values indicated an underestimation of observed data relative to the CMEMS MedFS data. This discrepancy may be partly attributable to the limited availability of deep-water observations during the winter season, which reduces the statistical robustness of the comparison. Furthermore, because no independent in situ calibration was performed, part of the observed BIAS values may reflect sensor-related uncertainties, particularly at greater depths.

The ability of the observed data to reproduce the vertical temperature patterns decreased in the intermediate layers (15–30 m and 30–50 m), where uRMSE values approached 2 °C during summer. This highlights the difficulty of resolving seasonal thermocline gradients in these layers, particularly during periods of strong stratification, and points to potential limitations in the capacity of turtle-borne data to fully capture vertical mixing processes and interactions between intermediate and deeper water masses.

To assess the effectiveness of observed data in capturing vertical temperature variations, a comparative analysis was conducted in the NA, MA, SA, and the WACC regions^38,39^. These areas, each characterized by unique oceanographic features, provided a suitable framework for evaluating the capability of satellite-tagged sea turtles to capture vertical temperature patterns under varying dynamical conditions. The SA, where surface waters interact with warmer intermediate layers and colder waters advected from the north, showed the best alignment between observed and CMEMS MedFS data, despite its complex circulation. In contrast, the agreement between the two datasets decreased in the NA and MA regions, which are strongly influenced by the Po River runoff and intense winter cooling driven by the Bora wind. The largest discrepancies were recorded in the WACC region, which hydraulically connects the gyres of the three sub-regions. Here, the relatively high uRMSE values, particularly at intermediate depths, may reflect limitations associated with the ~ 4 km spatial resolution of the CMEMS MedFS data, which is insufficient to resolve fine-scale coastal processes such as localized mixing, upwelling, and interactions with riverine inputs that can instead be detected by satellite-tagged sea turtles.

Future comparisons aimed at evaluating the ability of satellite-tagged turtle data to reproduce nearshore temperature profiles should be performed using unstructured grid models with higher spatial resolution, which could substantially improve temperature prediction accuracy in these dynamically complex areas.

Although the overlap between the observed data and ARGO float measurements was localized within a small area of the southern Adriatic Sea, the upper-ocean consistency check highlighted that observed data tended to overestimate ARGO float measurements down to approximately 8 m, whereas ARGO floats reported higher values below this depth. This inversion in the BIAS coincided with a sharp decline in the number of matched observations, suggesting that limited data availability at greater depths may affect the comparison.

Overall, our results suggest that satellite-tagged turtles can effectively resolve surface thermal patterns, providing valuable and complementary insights alongside conventional, higher-cost observing platforms within existing observing networks. The best agreement was found in the surface layer of the water column, likely due to the continuous assimilation of satellite data by the CMEMS MedFS model. In contrast, discrepancies between the observations and CMEMS MedFS data increased in deeper layers, reflecting the limited number of measurements and the infrequent temporal overlap with the tag acquisitions.

In conclusion, leveraging animal-borne sensors offers a promising opportunity to improve the acquisition of continuous and crucial information related to oceanic variables. Our results suggest that the observed data are able to reproduce key aspects of the vertical thermal structure, highlighting that sea turtle–borne observations can provide valuable insights in contexts where conventional observing systems have known limitations. Discrepancies between observed and CMEMS MedFS data were mainly located at greater depths, whereas near the surface, the observed data effectively captured seawater temperature patterns. Further studies comparing data acquired by animal-borne sensors and outputs from numerical models at finer coastal resolution would contribute to a more comprehensive understanding of the potential of these sensors for environmental research.

The results underscore the importance of data acquired by animal-borne sensors, highlighting their potential to enhance numerical ocean models when integrated with existing observational networks during the data assimilation process. However, to realize this potential, the deployed tags must undergo rigorous in situ calibration procedures both before and after deployment, covering the relevant sensor operating ranges to adequately address issues such as long-term sensor drift^12^. This study does not evaluate the impact of assimilating turtle-borne temperature data into numerical models and therefore does not quantify potential improvements in model performance.

Integrating these sensors with higher-accuracy temperature measurements could improve the detection of fine-scale thermal variability^40^. Furthermore, the addition of tri-axial accelerometers and gyroscopes would enable detailed analyses of animal movements during dives^41^ and, consequently, support more accurate estimates of their positions within the water column through the application of machine learning techniques^42^.

Although based on a limited number of tags, our results are nevertheless significant and confirm the value of sea turtles as data-collection platforms. Furthermore, these data could enhance the description of ocean processes (i.e., mixed layer properties) in poorly observed areas, highlighting the complementary role of turtle-borne observations within existing observing networks. Expanding tagging efforts in the future would unlock considerable potential, enabling broader and more detailed ocean coverage.

Overall, our findings pave the way for deeper exploration of the factors driving marine temperature variations using data collected by animal-borne sensors. Finally, our results confirm the potential of integrating animal-borne sensors into standard ocean monitoring frameworks, especially in regions with limited or logistically challenging observational coverage, while also providing important insights for advancing our understanding of underwater dynamics.

## Methods

### Study area

**Fig. 10:**
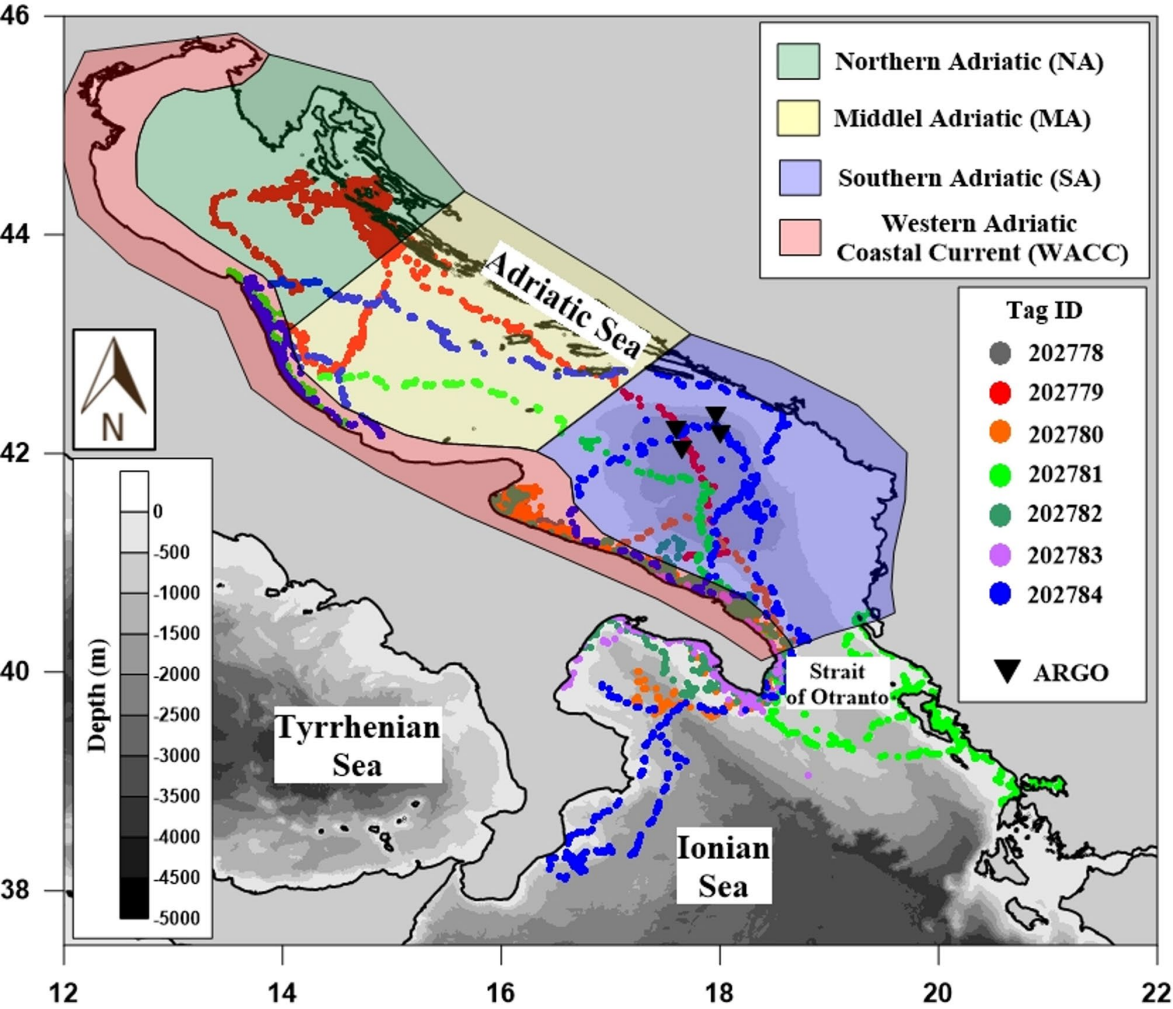
Study area and *C. caretta* routes. The map (generated using Surfer 9, Golden Software) displays the routes of seven loggerhead sea turtles in geographical coordinates; each individual route is identified by a unique color. Black triangles indicate the locations of ARGO float measurements. (Turtle route data adapted from^35^.

The study area comprehends the waters of the Adriatic and northern Ionian Seas (Fig. 10). The Adriatic Sea, bounded by the Italian Peninsula to the west and the Balkan Peninsula to the east, extends southward to the Strait of Otranto, where it reaches a maximum depth of 1,270 m^43^. Water exchange with the Ionian Sea occurs at the Strait of Otranto^54^. The northern Ionian Sea lies south of the strait and is characterized by a steeper continental slope than the Adriatic Sea with an offshore maximum depth of about 3,500-3,700 m^44^.

The general circulation of the Adriatic Sea is cyclonic and exhibits significant seasonal variability^38,39,45^. The climatological circulation pattern consists of well-known currents and gyre structures^33,44,45^. The three main gyres (the Southern, Middle, and Northern Adriatic Gyres^39^) are interconnected by two coastal currents, with seasonally varying characteristics. One current, the Western Adriatic Coastal Current (WACC), flows southward along the Italian coast from the Po River delta to the Strait of Otranto. The other, the Eastern Southern Adriatic Current (ESAC), flows northward from the Strait of Otranto along the eastern coast, reaching the central Adriatic^46^.

The northern Ionian Sea circulation is characterized by the interaction of various water masses, including Atlantic Water, Levantine Intermediate Water, and Adriatic Deep Water, which exits through the Strait of Otranto and renews the deep Ionian layers^47^. Surface circulation variability is influenced by the Adriatic-Ionian Bimodal Oscillating System (BiOS), a mechanism by which the deep thermohaline cell of the eastern Mediterranean, originating in the southern Adriatic, is connected to the upper circulation of the Northern Ionian Gyre (NIG) via positive feedback. This interaction causes decadal reversals in current direction that affect regional salinity and water mass distribution^48^. The Adriatic and northern Ionian Seas are among the most studied regions of the Mediterranean due to their complex oceanographic features, such as strong interactions between river inputs, dense water formation, and seasonal variability^49,50^, as well as their sensitivity to climate change and anthropogenic impacts, particularly in the shallow northern part^51,52^.

### Sea turtles tagging and reintroduction in the marine environment

Sea turtles were inadvertently caught by fishing equipment in the coastal regions of Puglia (Italy) and were subsequently rescued by the Management Consortium of Torre Guaceto (Carovigno, BR, Italy). Argos satellite tags (KiwiSat^®^ Glue On Series^53^) were applied on seven specimens of *C. caretta* (adults and subadults, Curved Carapace Length - CCL - between 50 and 70 cm) using epoxy adhesive at the “Luigi Cantoro” Sea Turtle Rescue Center. The tags were designed with specific features, such as low weight, compact shape, and abrasion resistance, making them suitable for attachment to animals such as the loggerhead sea turtle. These devices were capable of recording and transmitting geolocation data (date, UTC, latitude, longitude) together with selected environmental parameters collected during dives of the tagged specimens. Specifically, the satellite transmitters were equipped with pressure sensors for water depth (accuracy ± 1% of full scale) and temperature sensors for seawater temperature (accuracy ± 0.2 °C) according to the manufacturer’s specifications. No additional sensor calibration was performed. Subsequently, the animals were reintroduced into the marine environment (Table 2).

Ethical approval for turtle management was not required in this study, in accordance with local legislation and institutional requirements. All procedures were carried out by the specialized staff of the Marine Turtle Recovery Centre of Torre Guaceto. All experiments were conducted in compliance with the relevant national guidelines (ISPRA Manuals and Guidelines 89/2013^54^).

The “Luigi Cantoro” Sea Turtle Rescue Center, managed by the Torre Guaceto Management Consortium, operates under a special authorization pursuant to Presidential Decree 357/95, granted by the Ministry of the Environment (Official Register No. 0027230 of 27/12/2016).

This study is performed in accordance with relevant guidelines and regulations. All methods are reported in accordance with ARRIVE guidelines^55^.

### Observational dataset description

Vertical temperature profiles were reconstructed using data collected from satellite tags (observed data) deployed on seven marine turtles. Data storage was constrained by predefined sampling intervals and onboard memory limits, including a maximum storable dive duration of 240 min. In this study, vertical profiles were reconstructed exclusively from individual depth–temperature pairs rather than from binned summary data; however, sampling configuration, binning options, and memory constraints determine the effective vertical and temporal resolution of the dataset, and variations in these technical settings may influence the applicability of transmitter-derived observations, particularly when resolving fine-scale thermal stratification. These devices record information for each dive cycle and transmit the data once the animals resurface, via the Lotek web service, which relies on the Argos satellite positioning system.

Each dive record includes the geographic position of the individual, the associated UTC reference time, and a set of supplementary variables, such as Location Quality (LQ) and diagnostic information related to the tag’s electrical system (e.g., battery status). The LQ parameter comprises several classes (Z, B, A, 0, 1, 2, 3), corresponding to different levels of positional precision^56^. Additionally, each dive contains a sequence of paired seawater depth and temperature measurements, which form the basis for reconstructing vertical thermal profiles. Depth–temperature pairs were organized into data matrices representing 30 min of observations collected at 5-minute intervals. The number of records per dive depends on the duration of submergence and cannot exceed 240 min, after which the tag begins overwriting stored data. To enable temporal reconstruction of each depth–temperature measurement, the system records the elapsed time (in minutes) prior to satellite transmission at the end of the dive (DTtime); this information is stored at the end of each record. At the beginning of each record, the sea surface temperature (SST), measured when the turtle is near the surface, is reported along with the corresponding elapsed time before transmission (SSTtime).

The processing of turtle-borne tag data was designed to extract, reorganize, and reconstruct vertical seawater temperature profiles as a function of depth and time, producing a structured output dataset suitable for subsequent analyses.

First, rows containing diagnostic tag information (e.g., battery-related data) were removed. For each dive, position, date, and time were retained, keeping only valid observations associated with geographic coordinates (latitude and longitude) and LQ. Given the spatial resolution of the numerical model used in this study (4–5 km), only data assigned to LQ class 0 were included, as this class provides a positional precision estimated at approximately 1,500 m, which was considered consistent with the model’s grid scale.

Continuous temporal sequences were then identified to distinguish individual dive events. For each dive, temperature and depth measurements were reordered by reversing their temporal sequence (from maximum depth toward the surface) to allow correct reconstruction of the vertical profile. The DTtime variable was examined to detect potential temporal inconsistencies; when such inversions were identified, the profile was truncated at the first temporally coherent point to ensure internal consistency.

Starting from the UTC time associated with the turtle’s geographic position, the acquisition times of individual depth levels were reconstructed retroactively by progressively subtracting the recorded elapsed time intervals.

To exclude inaccurate observations, a comprehensive filtering process was applied to all reconstructed dives. Dives lacking the correct structural attributes were removed, specifically those without discernible start or end points or missing recorded values for time or depth. Finally, all data collected during the turtles’ surfacing (corresponding to a depth of 0 m) were discarded, as the sensor might have measured air temperature instead.

Each observation represents a unique combination of latitude, longitude, depth (later organized into different depth ranges, ranging from the surface down to the maximum depth of 175 m reached by the sea turtles), and day (from which seasonal information was derived). Table 3 reports the percentage of observations employed in the study, categorized by season and depth range.

**Table 3:** The percentage of observations employed in this study across different ranges of depths (0–15 m, 15–30 m, 30–50 m, and 50–100 m) across the seasons (winter, spring, summer, and autumn).

Season	Depth (m)				
	0-15 (%)	15-30 (%)	30-50 (%)	50-100 (%)	Total (%)
Winter	10.86	2.25	0.34	0.26	13.70
Spring	17.65	1.75	0.54	0.45	20.40
Summer	40.51	7.11	2.18	2.08	51.87
Autumn	9.30	3.36	0.68	0.69	14.02
*Total*	78.32	14.48	3.73	3.47	**100**

To assess the ability of the observed data to represent ocean thermal structures, a comparison with the CMEMS MedFS data was made. For each dive event, the observed data were matched to the nearest model grid point in space and time. When multiple observed profiles corresponded to the same model grid point and time step, the temperature profiles reconstructed from tag data were first averaged and then interpolated onto the model’s vertical depth levels. Observed and modeled profiles were then vertically collocated and analyzed within predefined geographic areas. For each depth and season, the mean temperature, bias, and µRMSE were calculated.

### Model data description

The observed sea water temperature data from satellite-telemetered sea turtles (Observed data) were compared to those of the Copernicus Marine Service Mediterranean Analysis and Forecasting System (CMEMS MedFS data) which provides high-resolution physical ocean forecasts and analyses for the entire Mediterranean Sea (MEDSEA_ANALYSISFORECAST_PHY_006_013)^57^.

This product delivers daily, quality-controlled data generated by a two-way coupled wave–current modelling system named MedFS. The ocean component is based on the NEMO (Nucleus for European Modelling of the Ocean^58^) model, while the wave dynamics are simulated using WaveWatch III^59^.

The model operates at a horizontal resolution of approximately 4.2 km (1/24°) and includes 141 vertical levels, capturing ocean conditions from the surface down to the seabed. It is driven by high-resolution atmospheric forcing provided by the European Centre for Medium-Range Weather Forecasts (ECMWF) at 1/10° horizontal resolution with 6 h temporal frequency. Freshwater inputs from 39 rivers are incorporated using runoff data from the European Flood Awareness System (EFAS-v5^60^), . Additionally, two lateral open boundary conditions are applied: one in the Atlantic Ocean and another in the Dardanelles Strait.

The system assimilates satellite altimeter data and in situ observations. In particular, the model assimilates in situ vertical profiles of temperature and salinity, mainly from ARGO floats and gliders, and, when available, from XBTs, as well as satellite Sea Level Anomaly along-track data. These data are assimilated using advanced data assimilation techniques (OceanVar^61^), which improve the accuracy of both the analyses and the 10-day forecasts. Satellite Sea Surface Temperature (SST) data are not directly assimilated but are instead used to correct the non-solar heat fluxes through a nudging technique.

Key physical variables provided by this product include sea temperature and salinity.

Models were validated and their quality assessment is available in the corresponding CMEMS Quality Information Document (available at: https://files.cmcc.it/Landing_pageOPA/Med%20sea/CMEMS-MED-QUID-006-013-V2.2.1.pdf).

The analysis focused on the upper 100 m of the water column, divided into four specific depth intervals (0–15 m, 15–30 m, 30–50 m, 50–100 m) selected to clearly represent the seasonal thermal structure. The maximum depth was set at 100 m due to the limited availability of data beyond this depth.

### Data analysis

Observed and CMEMS MedFS data were extracted and analyzed in MATLAB (R2024b). To thoroughly assess the comparison between the two datasets, it is essential to analyze their performance across a variety of metrics^62^. The Pearson correlation coefficient (r) was used to measure the linear correlation between observed and CMEMS MedFS data, providing insights into the degree of their alignment:

1 $$\:r=\frac{cov(x,y)}{{\sigma\:}_{x}{\sigma\:}_{y}}$$

where $\:cov(x,y)$ is the covariance, $\:{\sigma\:}_{x}$ is the standard deviation of $\:x$ and $\:{\sigma\:}_{y}$ is the standard deviation of $\:y$. Statistical significance was assessed at the 0.05 level.

The BIAS, representing the average difference between CMEMS MedFS and observed data, was computed as:

2 $$\:BIAS={\sum\:}_{i=1}^{N}\frac{\left({\stackrel{\prime }{y}}_{i}-{y}_{i}\right)}{N}$$

where $\:\stackrel{\prime }{{y}_{i}}$ is the corresponding i-th CMEMS MedFS data temperature, $\:{y}_{i}$ is the i-th observed data temperature and $\:N$ is the total number of data points. A positive BIAS indicates that CMEMS MedFS values are higher than the observed temperatures, while a negative BIAS indicates that the observed temperatures are higher than those of CMEMS MedFS.

The uRMSE was used as a metric to quantify the residual error attributable solely to variability, removing the effect of systematic bias, and is defined as:

3 $$\:uRMSE=\sqrt{{RMSE}^{2}-{BIAS}^{2}}$$

where RMSE is defined as:

4 $$\:RMSE=\sqrt{\frac{{\sum\:}_{i=1}^{N}{\left({\stackrel{\prime }{y}}_{i}-{y}_{i}\right)}^{2}}{N}}$$

In this study, the µRMSE and BIAS values are expressed as spatially and temporally averaged metrics computed over the entire dataset for specific vertical intervals. In the seasonal analysis, the temporal averaging was performed separately within each season, whereas in the spatial analysis the µRMSE and BIAS values were averaged within predefined Adriatic subregions.

To further evaluate the observational ability of satellite-tagged sea turtles’ data to capture ocean thermal structures, an upper-ocean consistency check was made using ARGO float measurements^63^ collected within the same geographic region and time frame (Fig. 9). Specifically, data from three ARGO floats (Platforms 6902848, 6903263, and 6903805) were obtained from the Euro Argo Data Selection (https://dataselection.euro-argo.eu/). To ensure statistical robustness in the calculation of BIAS across depths, only data pairs located within approximately 10 km of each other and acquired within ± 15 days were considered. Comparisons were performed at 1 m vertical intervals from the surface down to a depth of 12 m. Although the selected temporal window does not allow the resolution of daily temperature variability in the water surface layers, it provides a sufficiently large number of observations to enable a robust comparison between the observed data and other observational platforms.

## Data Availability

All data generated and analyzed in this study are available from the corresponding author (R. M.) on request.
